# Legislation Regarding the Insane Poor in France, in a Letter from Prof. C. A. Lee, M. D.

**Published:** 1850-02

**Authors:** C. A. Lee


ART. II.—*Legislation regarding the Insane Poor in France, in a Letter from* PROF. C. A. LEE, M. D.

PROF. FLINT, PARIS, Nov. 1st, 1849.

*Dear Sir*—Some of my leisure hours during the last month in Paris have been occupied in collecting information regarding the Insane Poor in France, and especially with respect to the legal provisions made in their behalf by the government. I have long been of opinion that the interests of this interesting class are too much neglected in our country, and that many abuses exist, connected with their management, which imperatively demand legislative interference. There is no class of patients so badly treated, so miserably neglected by our local Boards of Supervisors, and others whose duty it is to provide for their decent support and maintenance, as the insane poor of the United States, and if the present letter should have the effect, to awaken attention to this important subject, by showing what has been done and is doing in France, I shall be amply rewarded for my labor.

A law was enacted in 1848, directing the establishment of a Lunatic Hospital in each of the eighty-four Departments of France; a law which, however, has not yet been fully carried out. It appears from recent reports made to the French Assembly, that the number of the insane poor, in the different Departments of France is 12,286; viz. 5,935 male, and 6,354 females: for whose support during the last year, 4,826,168 francs ($965,-233) have been expended. The insane of France, as in our country, are treated in special Asylums, in Alms Houses, where special quarters are assigned them, or in particular establishments. The ratio of their allotment is as follows: in 37 public asylums (not including the national one at Charenton) 6,060 patients; in 25 Alms Houses, 4,621; in 11 special establishments, 1,905; total, 12,286. These different establishments are situated in 61 Departments, and distributed as follows: twenty-two public asylums are situated in the principal towns, or capitals of the department; one in the chief town of an arondisement, and 14 in the chief towns of a canton or district;* making a total of 37 different public asylums. There are 18 Alms Houses in the chief towns of a department, in which insane poor are received; one in the chief town of an Arrondissement, and 14 in chief towns of a canton; making a total of 37 Alms Houses the recipients of the insane. There are four particular establishments in the chief towns of a Department; one in the chief town of an Arrondissement, and 6 in the chief towns of a canton, making a total of 11 particular establishments. Of these 12,286 insane poor, the department of the Seine includes 2,536, about one fifth of the whole number, showing very clearly the influence of a city life in causing insanity. The department of the Lower Seine has 410 poor insane; Bouches-du-Rhone, 358; the Rhone, 320; the North, 270; Calvados, 230; The departments of the Corse has 30; the High Alpes, 35; Indre, 80; Eastern Pyrenees, 35: these departments have the lowest, as the former have the greatest number; showing the influence of healthy locality, mountain districts, and a rural life, in diminishing the ratio of insanity. It is a somewhat singular fact, that these departments of France which are poor and have the least fertile soil, have a greater proportion of foundling children to support, and fewer insane than other departments. The number of insane females in France is greater than that of insane males, the department of the Seine offering the greatest difference; while that of the High Alps, the Corse, and Tarn-etGaronne, presents a contrary result.

---

* One of the subdivisions of a department.

*Table showing the expense of supporting the Insane poor, in the different establishments in the Departments of France.*

| Departments. | Towns in which there are Establishments, to which the Departments send their Insane. | Nature of the Establishment. | Total Expense. Francs. | C. |
|---|---|---|---|---|
| Ain, | Bourg, | Asylum, | 32.000 | |
| Aisne, | Montreuil, | House of Mendicity, | 60.625 | |
| Allier, | Ousset, | Alms House, | | |
| | Saint Gilles, | Asylum, | 50.177. | 50 |
| Alpes, (Low) | Aix, | Alms House, | | |
| | Avignon, | Asylum, | 13.680 | |
| Alpes, (High) | Saint Robert, | Asylum, | 12.000 | |
| Ardeche, | St. Marie-de-Privas, | Maison de Sante, | 18.030 | |
| Ardenes, | Armentieres, | Asylum, | | |
| | Mareville, | Asylum, | 26.697.125 | |
| Aviege, | St. Liziers, | Asylum, | 33.400 | |
| Aub, | Mareville, | Asylum, | | |
| | St. Dizier, | Asylum, | 28.948 | |
| Aude, | Limoux, | Maison de Sante, | 21.114. | 50 |
| Aveyron, | Rhodes, | Alms House, | | |
| | St. Alban, | Alumx, | 22.700 | |
| Bouch-du-Rhone, | Aix, | Alms House, | | |
| | Marseille, | Asylum, | 155.174 | |
| Calvados, | Bon-Sauveur-de, Caen, | Maison de Sante, | 80.900 | |
| Coutal, | Aurillae, | Asylum, | 19.354 | |
| Charente, | Angouleme, | Alms House, | 19.344 | |
| Charente-Infer, | Lafond, | Asylum, | 48.125 | |
| Cher, | Bourges, | House of Mendicity, | 18.650 | |
| Covreze, | Lacellette, | Maison de Sante, | | |
| | Leyme, | Maison de Sante, | 13.000 | |
| Corse, | Marseille, | Asylum, | 13.800 | |
| Cote-d'Or, | Dijon, | Asylum, | 32.850 | |
| Cotes-du-nord, | Riom, | Alms House, | | |
| | Lacelette, | Maison de Sante, | 33.785 | |
| Creuse, | Lehon, | Maison de Sante, | 14.300 | |
| | St. Brieue, | Alms House, | 22.300 | |
| Dordogne, | Leyme, | Maison de Sante, | 22.300 | |
| Doubs, | Dole, | Asylum, | | |
| | Bellevaux, | Asylum, | 16.322. | 80 |
| Drome, | Avignon, | Asylum, | | |
| | St. Alban, | Asylum, | | |
| | La Guillotiere, | Maison de Sante, | 27.475 | |
| Eure, | Evreaux, | Alms House, | | |
| | Bon-Sauveur-de, Caen, | Maison de Sante, | | |
| | St. You, | Asylum, | 53.259 | |
| Eure-et-Loir, | Orleans, | Alms House, | | |
| | St. You, | Asylum, | 51.650 | |
| Finisterre, | Quimper, | Asylum, | | |
| | Morlaix, | Alms House, | 61.525 | |
| Gard, | St. Jean-de-Dieu, | Maison de Sante, | | |
| | St. Alban, | Asylum, | 34.810 | |
| Garonne, (High) | Lagrave, (Toulouse) | Alms House, | 57.487. | 50 |
| Gers, | Auch, | Asylum, | 22.090 | |
| Gironde, | Bordeaux, | Asylum, | | |
| | Cadillae, | Asylum, | 93.238 | |
| Herault, | Montpellier, | Alms House, | 65.420 | |
| Ille-et-Vilaine, | St. Meen, | Asylum, | | |
| | St. Malo, | Alms House, | 73.000 | |

*Table showing the expense of supporting the Insane poor, in the different establishments in the Departments of France.*

| Departments. | Towns in which there are establishments, to which the Departments send their Insane. | Nature of the Establishment. | Total expense. Francs. | C. |
|---|---|---|---|---|
| Indre, | Lacellette, | Maison de Sante, | | |
| | St. Marie, | Maison de Sante, | 88.275 | |
| Indre-et-Loire, | Tours, | Alms House, | | |
| | Orleans, | Alms House, | 36.900 | |
| Isere, | Saint-Robert, | Asylum, | 52.500 | |
| Jura, | Dole, | Asylum, | 23.468. | 75 |
| Landes, | Pau, | Asylum, | 11.250 | |
| Loir-et-Cher, | Blois, | Asylum, | | |
| | Orleans, | Alms House, | 35.439 | |
| Loire, | St. Jean-de-dieu, | Maison de Sante, | | |
| | St. Alban, | Asylum, | 45.925 | |
| Loire, (High) | St. Alban, | Asylum, | | |
| | Aurillae, | Asylum, | 16.400 | |
| Loire, (Lower) | Nantes, | Alms House, | 70.943. | 50 |
| Loiret, | Orleans, | Alms House, | 38.800 | |
| Lot, | Leyme, | Maison de Sante, | 17.800 | |
| Lot-et-Garonne, | Cadillae, | Asylum, | 22.662 | |
| Logere, | Lacellette, | Maison de Sante, | | |
| | St. Alban, | Asylum, | 11.200 | |
| Maine-et-Loire, | Angus, | Alms House, | | |
| | Saumer, | Alms House, | | |
| | Sarute Gaumes, | Asylum, | 62.060 | |
| Mauche, | Pontorson, | Asylum, | | |
| | Saint-La, | Alms House, | 35.720 | |
| Marne, | Chalons, | Asylum, | 66.774 | |
| Marne, (High) | St. Dizier, | Asylum, | 33.820 | |
| Mayenne, | Lorechegandon, | Maison de Sante, | 33.250 | |
| Manthe, | Mareville, | Asylum, | 60.694 | |
| Meuse, | Taius, | Asylum, | 47.650 | |
| Morbihan, | Lehon, | Maison de Sante, | | |
| | Vannes, | Alms House, | 48.000 | |
| Moselle, | Mareville, | Asylum, | | |
| | Fains, | Asylum, | 35.500 | |
| Nuvre, | La Charite, | Asylum, | 35.887 | |
| Nord, | Armentieres. | Asylum, | | |
| | Lille, | Asylum, | 118.560 | |
| Oise, | Clermont, | Maison de Sante, | 40.115. | 50 |
| Orne, | Alencon, | Asylum, | 58.940 | |
| Pas-de-Calais, | Saint-Venant, | Asylum, | 47.950 | |
| Puy-de-Dome, | Lacellette, | Maison de Sante, | | |
| | Riom, | Alms House, | 27.000 | |
| Pyrenees, (Low) | Pau, | Asylum, | 21.170 | |
| Pyrenees, (High) | Pau, | Asylum, | 13.178. | 50 |
| Pyrenees-Oriental | Perpignan, | Alms House, | | |
| | Limoux, | Maison de Sante, | 15.150 | |
| Rhin, (Low) | Stephansfeld, | Asylum, | 57.266. | 25 |
| Rhin, (High) | Stephansfeld, | Asylum, | 37.977. | 50 |
| Rhone, | L'Antigraaille, | Alms House, | 98.000 | |
| Saone, (High) | Mareville, | Asylum, | 24.525 | |
| Saone-et-Loire, | Bourg, | Asylum, | 35.500 | |
| Sarthe, | Le Mans, | Asylum, | 49.525 | |
| Seine, | Bicetre, | Alms House, | | |
| | La Salpetriere, | Alms House, | 1.295.394 | |
| Seine, (Lower) | St. You, | Asylum, | 184.500 | |
| Seine-et-Marne, | Clermont, | Maison de Sante, | | |
| | St. Vincent, | Asylum, | 35.650 | |

*Table showing the expense of supporting the Insane poor, in the different establishments in the Departments of France.*

| Departments. | Towns in which there are establishments, to which the Departments send their Insane. | Nature of the Establishment. | Total expense. Francs. | C. |
|---|---|---|---|---|
| Seine-et-Oise, | Clermont, | Maison de Sante, | 62.176. | 75 |
| Sevres, (Deux) | Niort, | Alms House, | | |
| | Leyme, | Maison de Sante, | 33.150 | |
| Sommes, | Armentieres, | Asylum, | | |
| | Lille, | Asylum, | 61.915. | 50 |
| | Clermont, (Oire) | Maison de Sante, | | |
| Tarn, | Bon-Sauveur, | | | |
| | A'Alby, | Maison de Sante, | 27.310 | |
| Var, | Aix, | Alms House, | 39.305 | |
| Varne-et-Garonne, | Montaubon, | Asylum, | 16.300 | |
| | Saint Remy, | Maison de Sante, | | |
| Vaucluse, | Avignon, | Asylum, | 32.950 | |
| Vendee, | Fontenay, | Alms House, | | |
| | St. Jacques de Nantes, | Alms House, | 26.300 | |
| Vienne, | Poitieres, | Alms House, | 29.200 | |
| Vienne, (High) | Lemoges, | Asylum, | 39.050 | |
| Vosges, | Mareville, | Asylum, | | |
| | Stephansfeld, | Asylum, | 33.439 | |
| Yonne, | Auxerre, | Asylum, | 67.525 | |
| | | | 4.826.188. | 70 |
| Seine, | Charenton, | Maison National, | 459.857 | |
| Total, | | | 5.286.025. | 75 |
| Or, | | | $1.059.205 | |

*Table showing the distribution of the Insane Poor in the Public and Private Asylums, and Alms Houses of France.*

| Departments. | Communes. | Number of Insane in Public Asylums. | Alms Houses. | Private Asylums. | Total. | Population. |
|---|---|---|---|---|---|---|
| Ain, | Bourg, | 254 | — | — | 254 | 346.188 |
| Aisne, | Montreuil Sous Laon, | 150 | — | — | 150 | 527.085 |
| Allier, | Saint Gilles, | 180 | 15 | — | 195 | 309.270 |
| | Cupet, | — | — | — | — | — |
| Ardeche, | Privas (Sainte Marie) | — | — | 83 | 83 | 353.782 |
| Ariege, | Sains Liziers, | 125 | — | — | 125 | 260.536 |
| Aube, | Limoux, | — | — | 56 | 56 | 282.882 |
| Aveyron, | Rhodes, | — | 75 | — | 75 | 370.951 |
| Bouch du Rhone, | Aix, | — | 103 | — | 103 | 362.323 |
| | Marseille, | 330 | — | — | 330 | — |
| Calvados | Caen, | — | — | 250 | 250 | 501.775 |
| Cantal, | Ausillae, | 123 | — | — | 123 | 262.117 |
| Charente, | Angonleme, | — | 26 | — | 26 | 365.126 |
| Charent, (Infer) | Lafont lez la Rochelle, | 125 | — | — | 125 | 449.639 |
| Cher, | Bourges, | 50 | — | — | 50 | 276.853 |
| Correze, | Lacellette, | — | — | 134 | 134 | 302.433 |
| Cote d'Or, | Dijon. | 90 | — | — | 90 | 385.624 |
| Cotes du Nord, | Lehon, | — | — | 122 | — | 605.563 |
| | St. Brienne, | — | 104 | — | 226 | — |
| Eure, | Evreux, | — | 80 | — | 80 | 424.762 |
| Finistere, | Morlaix, | — | 119 | — | — | 546.955 |

*Table showing the distribution of the Insane Poor in the Public and Private Asylums and Alms Houses of France.*

| Departments. | Communes. | Number of Insane in Public Asylums. | Alms Houses. | Private Asylums. | Total. | Population. |
|---|---|---|---|---|---|---|
| | Quimper, | 128 | — | — | 247 | — |
| Garonne, (High) | Toulouse , | — | 205 | — | 205 | 454.727 |
| Gers, | Auch, | 80 | — | — | 80 | 312.882 |
| Gironde | Bordeux, | 141 | — | — | — | 555.809 |
| | Cadillae, | 154 | — | — | 295 | — |
| Hesault, | Montpellier, | — | 168 | — | 168 | 357.846 |
| Ille et Vilaine, | Rennes, | 219 | — | — | — | 547.249 |
| | St. Malo, | — | 21 | — | 240 | — |
| Indre et Loire, | Tours, | — | 100 | — | 100 | 306.366 |
| Isere, | St. Robert, | 138 | — | — | 138 | 588.660 |
| Jura, | Dole, | 137 | — | — | 137 | 316.734 |
| Loineet Cher, | Blais, | 68 | — | — | 68 | 244.043 |
| Loire, (Lower) | Nantes, | — | 170 | — | 170 | 470.768 |
| Loiret, | Orleans, | — | 230 | — | 230 | 316.189 |
| Lot, | Leyme, | — | — | 147 | 147 | 187.603 |
| Lozere, | St. Alban, | 160 | — | — | 160 | 141.733 |
| Maine et Loire, | St. Gerume, | 109 | — | — | — | 477.270 |
| | Angers, | — | 33 | — | — | — |
| | Saumur, | — | 20 | — | 153 | — |
| Mouche, | Pontorgon, | 79 | — | — | — | 597.334 |
| | St. Lo, | — | 15 | — | 94 | — |
| Marne, | Chalons, | 166 | — | — | 166 | 356.632 |
| Maine, (Haute) | St. Dizier, | 112 | — | — | 112 | 255.969 |
| Mayenne, | Laroche, | — | — | — | — | 361.765 |
| | Gandon, | — | — | 110 | 110 | — |
| Meurthe, | Mareville, | 395 | — | — | 395 | 444.603 |
| Meuse, | Fains, | 224 | — | — | 224 | 326.372 |
| Morbihan, | Vannes, | — | 102 | — | 102 | 446.331 |
| Nievre, | La Charite, | 130 | — | — | 130 | 297.550 |
| Nord, | Armentie, | — | — | — | — | 1.026.417 |
| | Us, | 178 | — | — | — | — |
| | Lille, | 225 | — | — | 403 | — |
| | Clermont, | — | — | 362 | 362 | 398.641 |
| Oise, | Alencon, | 156 | — | — | 156 | 443.688 |
| Orne, | St. Venant, | 180 | — | — | 180 | 685.645 |
| Pas de Calais, | Rione, | — | 145 | — | 145 | 587.566 |
| Pay de Dome, | Pau, | 113 | — | — | 113 | 451.683 |
| Pyrenees, (Low) | Perpignan, | — | 30 | — | 30 | 164.315 |
| Pyrenees, (East) | Stephansfeld, | 285 | — | — | 285 | 561.859 |
| Rhin, (Lower) | L'Antiquaille, | — | 320 | — | — | 482.024 |
| Rhone, | La Guillotiere, | — | — | 194 | 514 | — |
| | Le Mons, | 135 | — | — | 135 | 466.888 |
| Sarthe, | Bicetre, | — | 856 | — | — | 1.106.891 |
| Seine, | Salpetriere, | — | 1.500 | — | 2.536 | — |
| | St. You, | 457 | — | — | 457 | 780.725 |
| Seine, (Lower) | Niort. | — | 95 | — | 95 | 304.103 |
| Sevres, | Alby, | — | — | 74 | 74 | 351.656 |
| Tarn, | Montanbau, | 40 | — | — | 40 | 242.184 |
| Tarn et Garonne, | St. Remy, | — | — | 75 | 75 | 323.404 |
| Var, | Avignon, | 178 | — | — | 178 | 251.080 |
| Vaucluse, | Fontenay, | — | 90 | — | 90 | 356.453 |
| Vendie, | Poitieres, | — | 80 | — | 80 | 288.002 |
| Vienne, (High) | Limoges, | 170 | — | — | 170 | 293.000 |
| Youne, | Auxerre, | 183 | — | — | 183 | 362.962 |
| | Total, | 6.060 | 4.621 | 1.605 | 12.286 | |
| Seine, | Charenton, | | | | 467 | |
| Total. | | | | | 12.733 | |

The above does not include all the departments of France, but only those from which I have been able to obtain statistics from reports made during the last year to the Assembly.

From the data furnished in the above tables, the reader can easily calculate for himself the expense of supporting each patient in the different establishments in each department. For example, the department of Ain has 254 insane patients, who are supported at an annnal expense of 32,000 francs, which will give an average sum of 125 francs to each, or $35; and in this department they are supported in a public asylum. In the department of Aisne, where they are provided for in a house of mendicity, there are 159; whose support is estimated at 60,625 francs, or about $80 per head. In the department of Allier, in which there are 195 patients, supported in an alms-house, at an annual expense of 50,177 francs, or about $51 per head, annually. In the department of Ardeche, where ere are 83 insane, supported in a *Maison de Sante*, at an expense of 18,030 francs, annually, the average cost per head, is about $43 per annum. This may serve to indicate the relative expense of supporting the pauper insane in the different establishments provided for their reception in France.

Medical legislation in France, in behalf of the insane, dates from the year 1838. It was then ordained, that each department should have a special establishment for their reception and care; a wise regulation which has not yet been fully carried out. A proviso, however, was inserted that, temporarily, it is supposed, an arrangement might be effected for the same purpose, with some public or private establishment, either in or out of a particular department, this arrangement to be approved by the Minister of the Interior. These public institutions, devoted to the management of the insane, are placed under the direction of the public authority, while private institutions, of the same kind, are under its supervision, or inspection. The prefect, and the persons specially delegated for this purpose by the minister of the interior, the president of a tribunal, the public attorney, justice of the peace, or mayor of the district, are charged with the duty of visiting these public and private establishments. They are to receive the claims of patients admitted, and make, in their behalf, all enquiries necessary to learn their circumstances and condition. Public and private institutions are to be visited, at least once every three months, on indeterminate days, by the attorney general of the Arondissement. No one can establish an institution for the reception of the insane, without the authority of the government. Private establishments, devoted to the treatment of other maladies, are not allowed to receive insane patients, unless they are placed in apartments wholly separated from the others, and then a special authority from the government is necessary, which places them, like all the others, under its strict surveillance.

All the rules for the internal regulation of these establishments, whether wholly, or in part devoted to the management of the insane, must have the approval of the minister of the interior. The chiefs or responsible di-directors of these institutions for the insane, whether public or private, are not allowed to receive any patient afflicted with mental alienation, unless they are furnished, 1st, with an application for admission, containing the name, profession, age and residence, both of the applicant and the patient, and the degree of relationship which exists between them. The demand must be written and signed by him who presents it; and if he is unable to write, it must be approved by the mayor or commissary of police. The chiefs, overseers, or directors, must satisfy themselves, under their responsibility, of the individuality of the person making the application, when it has not been endorsed by the mayor or commissary of police.

2d. A certificate from some physician is required, stating the mental condition of the patient and pointing out the peculiarities of his malady, and the necessity of subjecting him to the treatment and restraint pursued in insane hospitals. A certificate is not received if it has been drawn up more than a fortnight before it is sent to the chief or director of the institution, or if it is signed by a physician attached to the establishment, or if the physician who gives it is the parent of, or is allied in the second degree inclusively, to the chiefs or proprietors of the establishment in which the patient is sought to be placed. In very urgent cases, such certificates however, are dispensed with.

3d. A passport, or some other document proving the identity of the patient, is also required. All these separate documents are then to be transcribed, and the copy sent within twenty-four hours, with a certificate of the physician of the establishment, to the prefect of police at Paris, to the prefect or sub-prefect of the chief town of the department or arondissement, and to the mayor in the other districts. The mayor or sub-prefect immediately transmits it to the prefect.

If the placement is made in a private establishment, the prefect, within three days after the reception of the above certificates, selects one or more physicians to visit the patient, designated for the purpose of ascertaining his mental condition, and making an immediate report. This physician may associate with him some other whom he may select. Within the same period the prefect must communicate the names, profession and residence, both of the patient and the person who makes the placement, as well as the causes of the same, 1st, to the State's attorney of the aron-disement in which the patient has his home. 2d. To the State's attorney of the arondisement in which the asylum is situated; and these regulations must be complied with, whether the institution be a public or private one.

Fifteen days after the placement of an insane patient in an establishment, public or private, a new certificate must be forwarded by the superintendent, (physician) confirming, or rectifying, if need be, the statements contained in the first certificate, indicating the return, more or less frequent, of the paroxysms or acts of insanity. Every establishment for the insane must contain a register, numbered and signed by the mayor, in which must be recorded the names, profession, age and residence of the patients, the record of the judgment of interdiction, if such has been pronounced, and the name of the guardian; the date of their placement, the names, profession and residence of the individual, whether parent, guardian, &c., who shall have demanded the admission. The register must also contain, 1st, the certificate of the physician, with the demand for admission. 2d. those which the physician of the establishment may have addressed to the authority, conformably with the articles above quoted. The physician is obliged to enter upon this register, at least once a month, the changes which may occur in the mental state of each patient, and the increase or diminution of the paroxysms. This register is to be exhibited to all persons who have a right, according to the regulations which are pointed out in the law, to visit the establishment; and such persons have a right to record upon the same register any observations or suggestions which they think proper to make. All patients admitted into insane establishments, are entitled to a discharge, whenever the phystcians shall record upon the register, that a cure has been effected; and if the patient is a minor or an interdict, then the opinion of the physicians must be immediately communicated to the persons interested, or the public attorney of the district—(*procureur du roi*). Even before the physicians have certified a cure, any patient placed in an insane institution, is entitled to a discharge, if his dismission is demanded by the guardian, husband, wife, or in the absence of these. ascending or descending relatives of the person who requested the admission; and in short, any person authorized by the family to ask a discharge. When any opposition is made, by relations or others, to the dismissal of a patient, the councillor of the family decides. Nevertheless, if the physician of the establishment is of opinion that the mental state of the patient will compromise public order, or the safety of individuals, previous information is to be furnished to the mayor, whose duty it is to order a temporary suspension to the dismissal, for the purpose of referring the case, within twenty-four hours, to the prefect. This provisional suspension, however, ceases after the expiration of fifteen days, if the prefect does not, within this period, give contrary orders, according to a provision of the law. This order of the mayor is to be transcribed upon the register. In case of minority or interdiction, the guardian alone can demand a patient's dismissal. The prefect can at any time order the dismissal of persons, voluntarily placed in an insane establishment. In any case, an interdict cannot be replaced, except by his guardian; and if a minor, except by the authority of those under whose charge he is placed by the law.

With respect to "placements" ordered by the public authority at Paris, and in the Departments, it is the duty of the prefects to order the "placement" in a lunatic establishment, of every person, whether an "interdict" or not, whose mental condition is likely to compromit public order, or the safety of individuals. The orders of the prefects must state the reasons, and circumstances which render the measure necessary; and these orders like all others, have to be inscribed upon the register, as above mentioned.

In case of imminent danger, as certified to by a physician or public notariety, the Commissaries of Police at Paris, and the Mayors in the other districts, must order, with respect to persons afflicted with mental aberration all necessary provisional measures, for the purpose of referring the case within twenty-four hours to the prefect, who must give it immediate attention.

The chiefs, directors, or responsible superintendents of public lunatic establishments in France, are obliged by law, to send in to the prefect, in the first month of each quarter, a report drawn up by the physician of the establishment, on the state of each patient, the nature of his malady, and the results of the treatment. The prefect then decides, respecting each individual, whether he shall be retained longer or discharged. With regard to persons who shall voluntarily have been placed in an insane establishment, and in cases where their mental condition might compromit public order, or the safety of individuals, the prefect may issue a special order to prevent patients from being discharged; and the chiefs, directors, and superintendents, are bound to conform to this order.

The public attorneys must be furnished with copies of all the orders above mentioned, which are forwarded to the mayor of the district, where the patient has his domicile; whose duty it is immediately to communicate the same to his friends. The Minister of the Interior must also be furnished with copies of the same. The discharge may be effected at any time, if the physicians of the establishment think such discharge advisable. Civil hospitals and alms houses, are obliged to receive temporarily, insane patients sent to them in conformity with the regulations above mentioned; and in all the communes, where hospitals or alms houses exist, they are bound to receive the insane, and in places where they do not exist, the mayors must provide a place for their reception, whether it be a private dwelling or house rented for the purpose. In no case is it allowed to shut up the insane with criminals, or the accused, nor lodge them in any prison whatever; and these regulations apply to all insane, who are ordered by public authority to some public or private establishment.

*(To be continued.)*

